# Five-component, one-pot synthesis of an electroactive rotaxane comprising a bisferrocene macrocycle

**DOI:** 10.3762/bjoc.16.128

**Published:** 2020-06-30

**Authors:** Natalie Lagesse, Luca Pisciottani, Maxime Douarre, Pascale Godard, Brice Kauffmann, Vicente Martí-Centelles, Nathan D McClenaghan

**Affiliations:** 1 Institut des Sciences Moléculaires, CNRS UMR 5255, University of Bordeaux, Talence, France; 2 Institut Européen de Chimie et Biologie, CNRS UMS 3033, INSERM US001, University of Bordeaux, Pessac, France

**Keywords:** ferrocene, macrocycle, rotaxane, single crystal X-ray structure, template

## Abstract

The templated clipping of a ferrocene-grafted isophthalic acid derivative to encircle a hydrogen-bonding axle through the reaction with 1,4-bis(aminomethyl)benzene is described. The constituent electroactive macrocycle of the resultant [2]rotaxane is a homologue of the versatile benchmark tetraamide variant developed by Leigh and co-workers. The relative templating effect of different hydrogen-bonding motifs in rotaxane and pseudorotaxane generation is compared, with yields varying from 0 to 41%. The electrochemical properties and single crystal X-ray structure of a doubly ferrocene-decorated [2]rotaxane are further reported.

## Introduction

**Figure 1:**
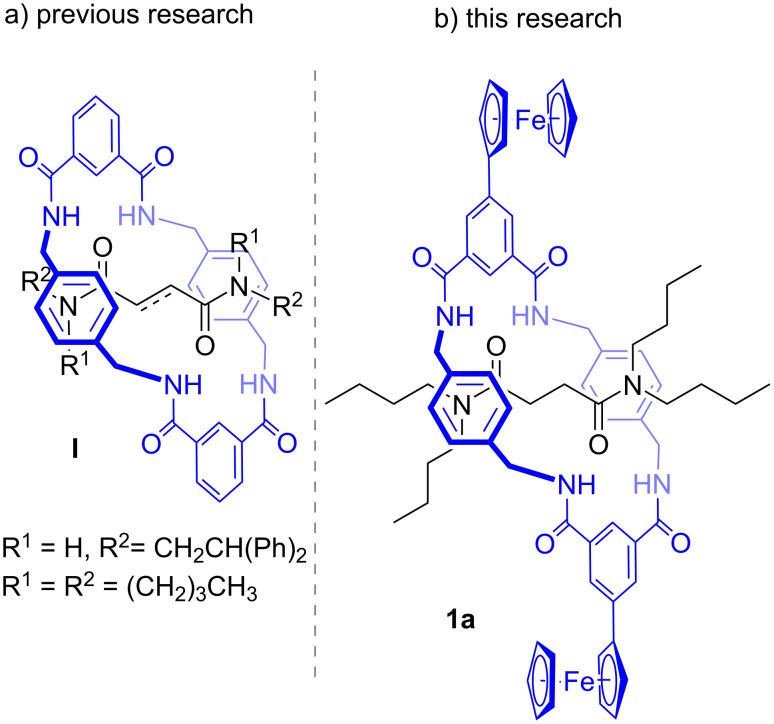
(a) Non-functionalized rotaxanes previously described in the literature. (b) The redox-active rotaxane developed in this work.

The development of interlocked molecules with tailored properties allowed the preparation of molecular machines able to perform several functions as artificial molecular switches [1]. The template-directed synthesis of such sophisticated catenane and rotaxane molecular architectures allowed the expansion of chemical diversity and properties. Among these architectures, electroactive rotaxanes have been described to act as stimuli-responsive molecular “shuttles” with potential applications to prepare nanoscale devices for computing and biomimetic engineering [2–3]. The highly efficient rotaxane formation developed by Leigh allowed the generation of a tetraamide macrocycle on a fumaramide or succinamide thread in high yields. This methodology consisted of a 4-component macrocyclization reaction, templated by the thread to obtain the corresponding interlocked molecule (Figure 1a) [4–5]. This class of macrocycle has proved extremely versatile, having given rise to a wealth of functional architectures [6–14]. In this context, we report the synthesis of a rotaxane, where a “clipping” reaction generates a tetraamide macrocycle with two peripheral ferrocene moieties on a preformed thread (Figure 1b). The resulting versatile and easily accessible electroactive macrocycle is anticipated to prove a valuable component for the construction of novel redox-active supramolecular systems, such as rotaxanes with strongly binding H-bonding templating sites, or indeed juxtaposed into existing functional architectures and benchmark variants.

## Results and Discussion

### Synthesis

**Figure 2:**
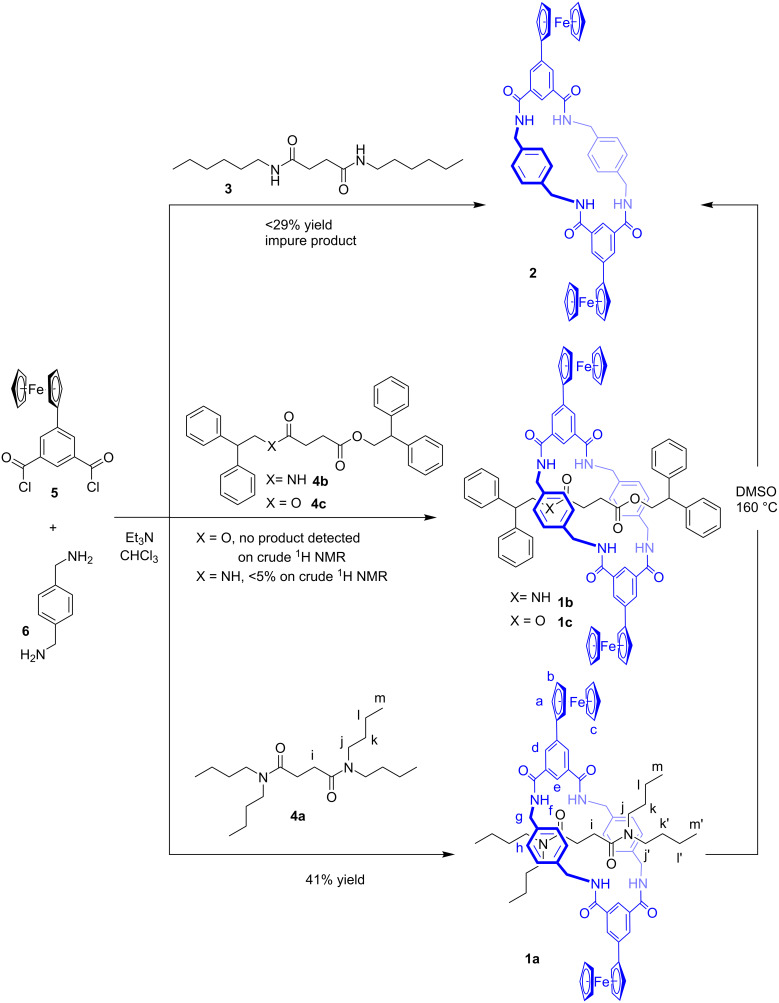
Synthesis of the redox-active rotaxanes **1** and macrocycle **2**.

Our first approach to the synthesis of macrocycle **2** was carried out using *N*,*N'*-dihexyl-1,4-butanediamide as the template, as represented in Figure 2. While the formation of some macrocyclic product was identified by ^1^H NMR and MS, it could not be completely separated from impurities (<29% yield after chromatographic purification). The isolated material had low solubility, which is likely due to self-aggregation via complementary amide hydrogen bonds. This prompted us to use a thread with bulky stopper groups to prepare the corresponding rotaxane compounds, which would increase the solubility by the formation of intramolecular macrocycle-thread hydrogen bonds, thereby reducing self-aggregation. To this end, a five-component clipping strategy was adopted using different tetrabutylsuccinamide threads (Figure 2) with varying hydrogen-bond basicity (amides > esters) [4,15]. Threads containing an ester group were selected as esters could potentially be hydrolysed post-clipping to allow isolation of the macrocycle. The diamide/bisamide thread had flexible stoppers, which may enable dethreading by slippage in a polar solvent as previously observed [5,16].

When the reaction was performed with the double ester template **4c**, it was not possible to identify any proton signals corresponding to the rotaxane in the crude ^1^H NMR (see Supporting Information File 1). The ester-amide template **4b** also proved inefficient in rotaxane formation, indeed only weak signals of the rotaxane were observed in the crude ^1^H NMR of the reaction mixture (<5% yield) and the product could not be isolated. Then, a more efficient template was employed with a double amide bonding moiety and the reaction of tetrabutylsuccinamide thread **4a** and *p*-xylylenediamine (**6**) and ferrocene isophthaloyl chloride **5** yielded the corresponding rotaxane **1a** in 41% yield. Unlike the free ferrocene macrocycle, the ferrocene rotaxane **1a** was soluble in CDCl_3_ suggesting a complementary macrocycle-thread hydrogen-bonding interaction. While the rotaxane **1a** was insoluble at room temperature in DMSO-*d*_6_, heating at 160 °C dissolved the rotaxane. Macrocycle dethreading in DMSO-*d*_6_ at 160 °C was monitored by ^1^H NMR (Figures S12 and S13 in Supporting Information File 1). After 1 h of heating at 160 °C, the integration of the NMR signals showed circa 58% of rotaxane **1a**, along with free macrocycle **2** and thread **4a**. After 23 h of reaction, no starting material remained and only 34% of the macrocycle was present, as judged by proton resonances in the ^1^H NMR spectrum, which along with the reaction mixture turning dark brown and the presence of a black precipitate suggested degradation.

**Figure 3:**
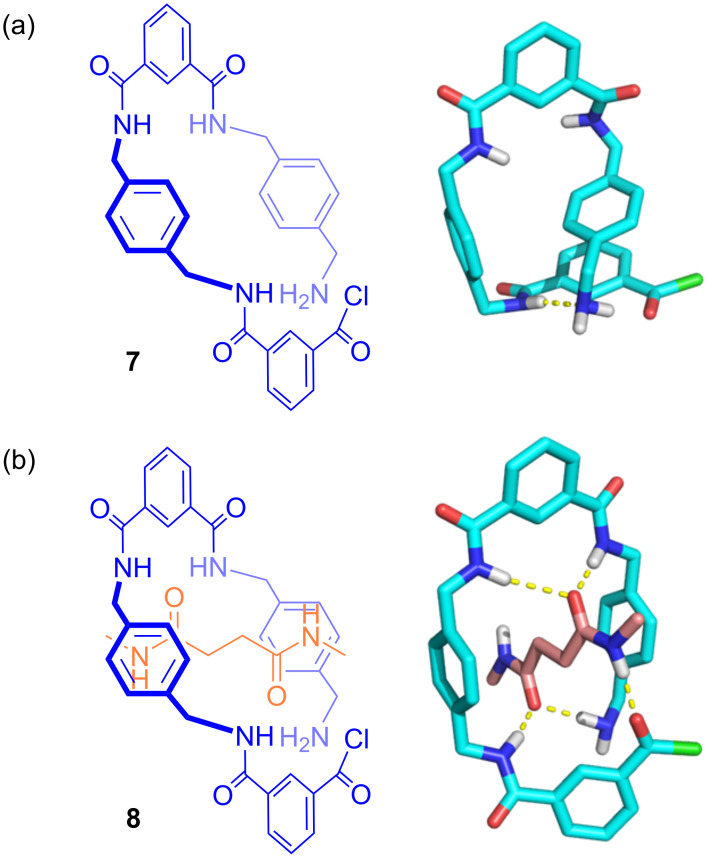
Most stable conformers obtained by Monte Carlo conformational search using model compounds. (a) Model macrocyclization reaction intermediate in the absence of a template; (b) model macrocyclization reaction intermediate in the presence of a template.

In order to evaluate the templating effect of the *N*,*N’*-dihexyl-1,4-butanediamide (**3**), molecular modelling studies of the 4-component macrocycle precursor were carried out. To reduce computational cost a model compound was considered, replacing the ferrocene by a hydrogen atom and replacing the hexyl chains of the template by methyl groups. Monte Carlo conformational searches were performed on the model structures (**7** and **8**) to obtain the most stable conformers (Figure 3) [17–18]. A comparison of the geometries of the most stable conformers indicated that the presence of the template produces a favourable preorganisation of the precursor, not only placing the amino and acid chloride groups in proximity, but also arranging the molecule in an appropriate reactive position favouring the intramolecular macrocyclization reaction (Figure 3b) [19]. Despite the theoretically favourable preorganization, the observed experimental templating effect was modest as the macrocyclization yield was only ≈29% of impure material. In contrast, in the absence of the template both reactive groups were not positioned in a geometry favouring macrocycle formation, which may prove conducive to oligomerization or catenane formation (Figure 3a).

**Figure 4:**
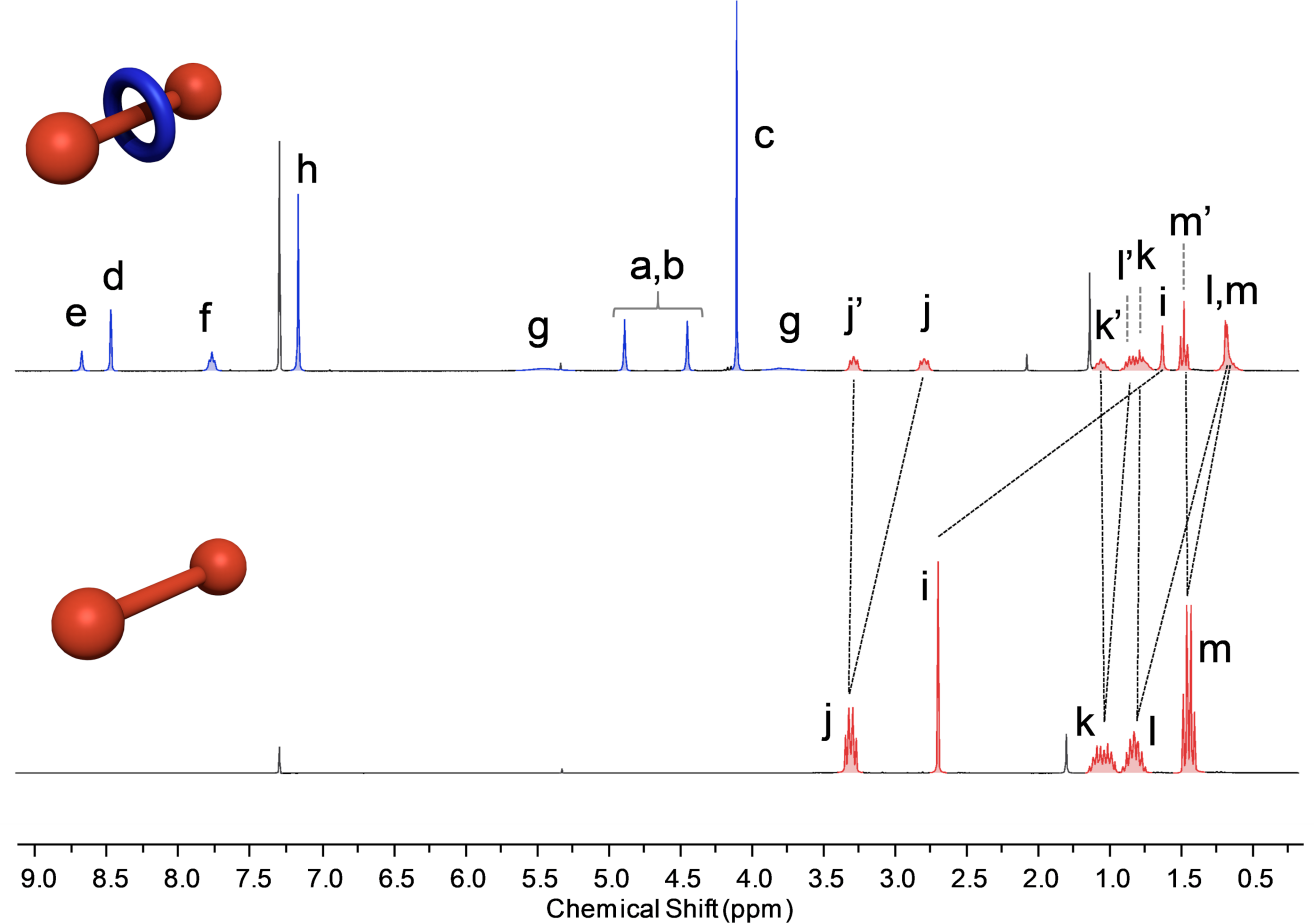
^1^H NMR spectrum (300 MHz) of rotaxane **1a** (top) and thread **4a** (bottom) in CDCl_3_ (a designation of the signals is described in Figure 2).

The ^1^H NMR spectrum of the rotaxane **1a** in CDCl_3_ (Figure 4) showed a characteristic upfield shift (1.57 ppm) of the succinic protons *i* by the macrocycle phenyl rings providing evidence of the interlocked nature of the structure. An analysis of the signal shapes showed a broadening (peak width of 55 Hz in the 300 MHz ^1^H NMR) of the macrocycle CH_2_ protons *g*, suggesting a dynamic process of macrocycle motion around the thread close to the coalescence temperature, in contrast with the other rotaxane proton signals, which had a peak width in the range of 2–5 Hz in the 300 MHz ^1^H NMR spectrum. A similar macrocycle pirouetting behaviour was observed in related 1-station [2]rotaxane systems [20]. An analysis of the thread signals in the ^1^H NMR of the rotaxane showed a non-equivalency of both butyl chains indicating a slow rotation of the amide bond in the NMR timescale (Figure 4). One of these butyl chains is in close proximity to the macrocycle aromatic rings producing a shielding of the signals (*j, k, l,* and *m*).

**Figure 5:**
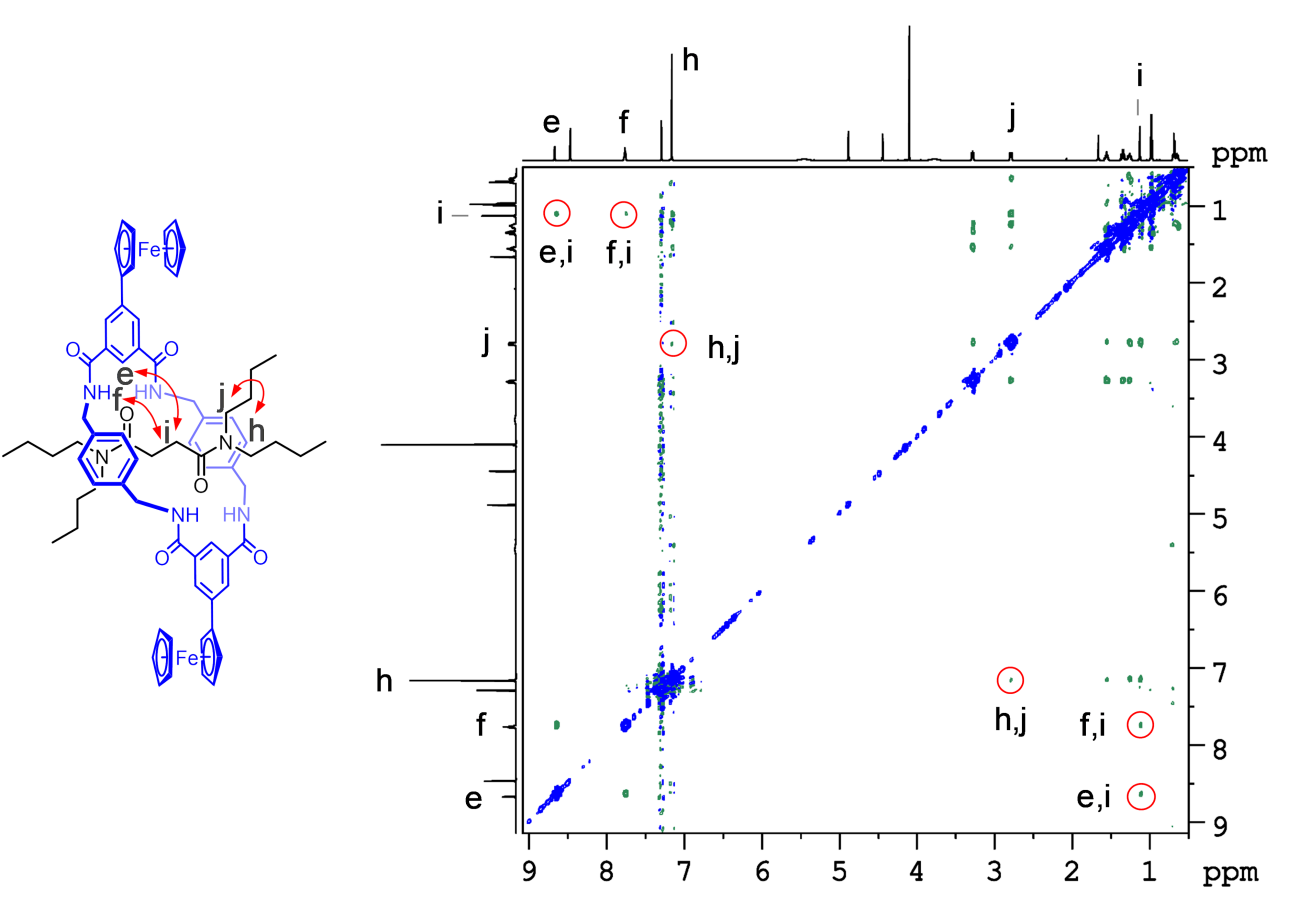
^1^H,^1^H-ROESY NMR spectrum (600 MHz) of the rotaxane **1a** in CDCl_3_.

Further evidence of the interlocked nature of rotaxane **1a** could be obtained from the ^1^H,^1^H-ROESY NMR spectrum. Multiple through-space cross-coupling correlation between the thread and the macrocycle protons were observed between the mechanically-bonded macrocycle and the thread (Figure 5). Additional proof of the mechanical bond was provided by ^1^H DOSY NMR showing the same diffusion for thread and macrocycle signals with a diffusion coefficient of −9.33 m^2^/s and a hydrodynamic radius of 8.7 Å (see Figure S4 in Supporting Information File 1).

### Electrochemistry

**Figure 6:**
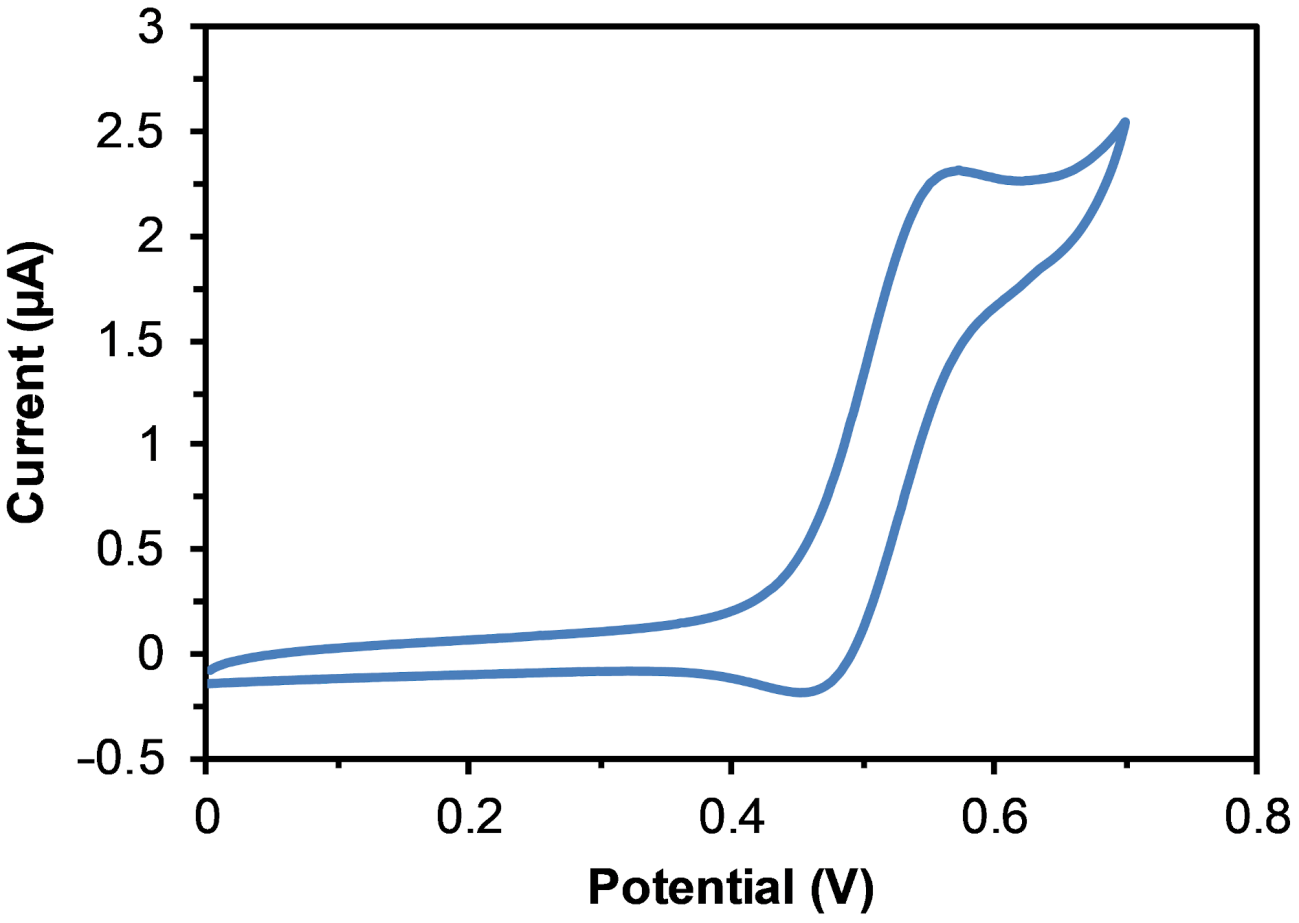
Cyclic voltammogram of ferrocene rotaxane **1a** (0.67 mM) in CH_2_Cl_2_/CH_3_CN 1:5 (TBAPF_6_ 0.10 M, scan rate = 10 mV/s).

The ferrocene-containing rotaxane **1a** was studied by cyclic voltammetry in CH_2_Cl_2_/CH_3_CN 1:5 (v/v) using a three-electrode cell, with a glassy carbon working electrode, a silver wire counter electrode and an Ag/AgCl (3 M KCl) reference electrode. Tetrabutylammonium hexafluorophosphate was used as the supporting electrolyte. Rotaxane **1a** presents a reversible redox transition at *E*_1/2_ = 0.51 V (that corresponds to *E*_1/2_ = +0.47 V vs SCE), where *E*_1/2_ = (*E*_p_^a^ + *E*_p_^c^)/2 (Figure 6). As only one oxidation wave was observed, this showed that the two metal centres were electronically decoupled, while the oxidation potential of ferrocene was *E*_1/2_ = +0.380 V vs SCE showing that rotaxane **1a** retained the redox properties of the parent ferrocene [21–22]. Importantly, the full reversibility of the one electron oxidation–reduction process attested to the stability of the electroactive system.

### Solid-state X-ray structure

**Figure 7:**
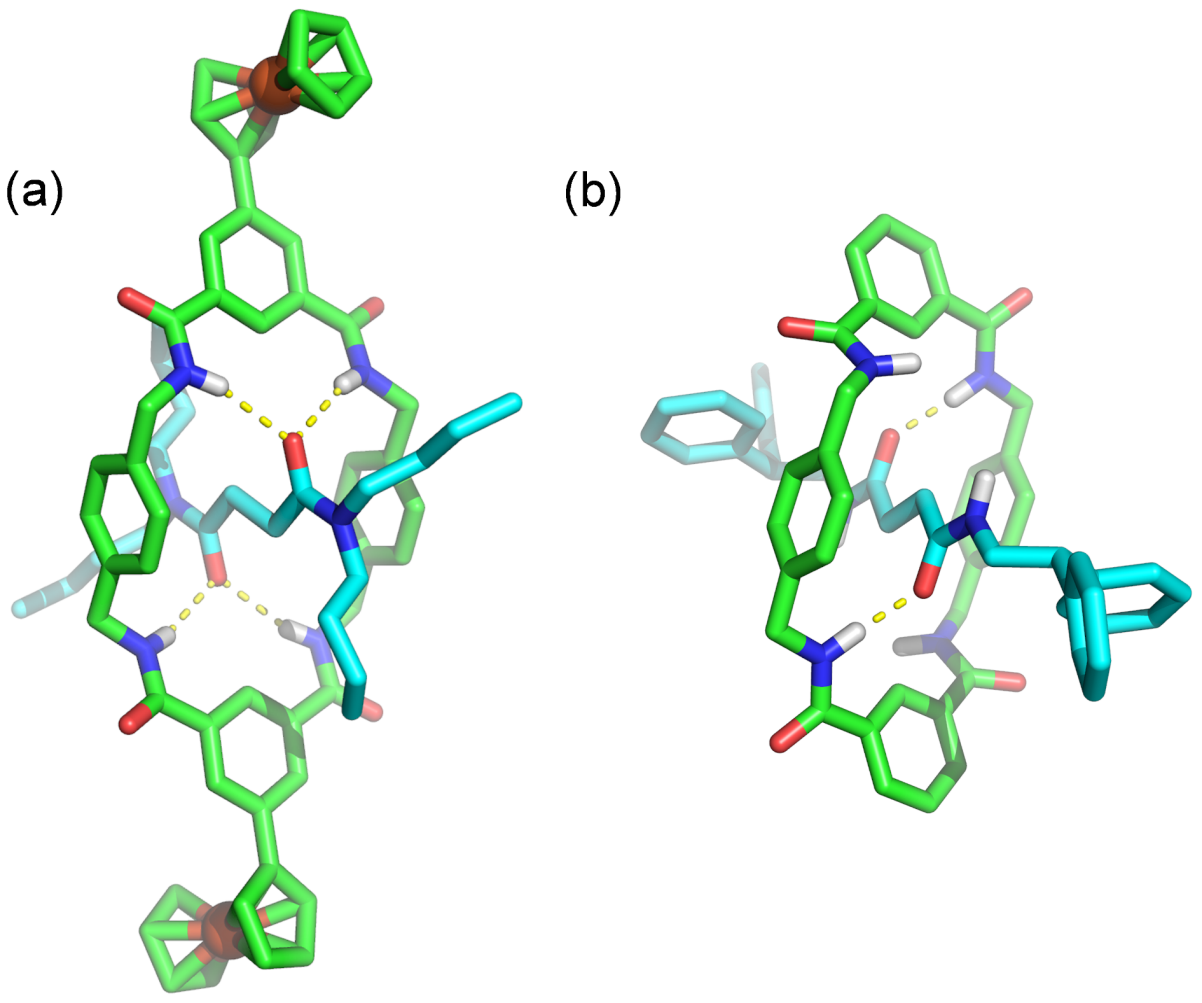
Single crystal X-ray structures of (a) rotaxane **1a** and (b) Leigh’s rotaxane **I** [4].

The solid-state structure of rotaxane **1a** was determined by single crystal X-ray diffraction of crystals obtained by slow evaporation of a dichloromethane solution. The analysis of the X-ray solid-state structure of the rotaxane **1a** showed a significant difference to Leigh’s rotaxane **I** [4]. Rotaxane **I** only presented two macrocycle–thread hydrogen bonds, with the other two macrocycle amides forming hydrogen bonds with the competitive crystallization solvent DMF. In contrast, rotaxane **1a** presented four thread–macrocycle hydrogen bonds (Figure 7 and Table 1). The strength of the hydrogen bonds could be estimated by the donor–acceptor distances: 2.2–2.5 Å “strong, mostly covalent”, 2.5–3.2 Å “moderate, mostly electrostatic”, and 3.2–4.0 Å “weak, electrostatic” [23]. The corresponding stabilization energies were estimated to be in the ranges 40–14 kcal/mol, 15–4 kcal/mol, and < 4 kcal/mol, respectively. The macrocycle conformation in the rotaxane placed the two ferrocene groups with a metal centre-to-metal centre distance of 20.7 Å.

**Table 1:** Selected H···O distances from the solid-state structures.

compound	donor–acceptor distance (Å)

Fc-rotaxane **1a**	3.04, 3.04 3.12, 3.12
Leigh rotaxane **I**	2.93, 2.93

## Conclusion

A five-component, one-pot reaction gave rise to an electroactive [2]rotaxane, through four amidination reactions between two ferrocene-grafted isophthalic acid derivatives and two 1,4-diaminomethylbenzene molecules, in the presence of a suitable hydrogen-bonding template. Among the different templates employed, **4a** proved to be the most successful giving rise to rotaxane **1a** in 41% yield. Meanwhile, **4b** and **4c** gave little or no rotaxane formation. Cyclic voltammetry showed that the electroactive ferrocene units were electronically decoupled and retained the reversible oxidation properties of the parent compound, while the single crystal X-ray structure of a doubly ferrocene-decorated [2]rotaxane indicated four relatively long and seemingly weak NH···CO hydrogen bonds. As the [2]rotaxane could be efficiently constructed in an analogous clipping fashion to the versatile benchmark tetraamide variant developed by Leigh and co-workers, one might anticipate that this doubly ferrocene-decorated motif could be directly transposed into a range of functional electroactive interlocked architectures.

## Experimental

### Materials and methods

All chemicals and solvents were obtained from commercial sources and used without further purification. Dry solvents were obtained using a standard solvent purification system, except for dry CHCl_3_, which was purchased from Sigma-Aldrich. Mass spectra and NMR spectra were performed in the CESAMO analytical facilities (Bordeaux, France).

NMR: ^1^H and ^13^C NMR spectra were recorded using a Bruker Avance 300 or a Bruker Avance III 600 spectrometer. Chemical shifts were reported in ppm and referenced to the residual solvent peaks. NMR chemical shifts (δ) were reported in parts per million (ppm) and coupling constants *J* are given in hertz (Hz). Multiplicity for each signal is indicated as follows: s = singlet, br s = broad singlet, d = doublet, t = triplet, and m = multiplet.

Mass spectrometry: Field desorption spectra (FD) were recorded on an AccuTOF (JEOL) mass spectrometer using an FD emitter with an emitter voltage of 10 kV. The sample (1–2 µL) was deposited on a 13 μm emitter wire. Electrospray spectra (ESI) were recorded on a Qexactive (Thermo) mass spectrometer. The instrument was equipped with an ESI source and spectra were recorded in the positive mode. The spray voltage was maintained at 3200 V and the capillary temperature set to 320 °C. Samples were introduced by injection through a 20 µL loop into a 300 µL/min flow of methanol from the LC pump.

Electrochemistry: Cyclic voltammetry was carried out in a Metrohm Autolab PGSTAT302N potentiostat using a three-electrode cell, with a glassy carbon working electrode, a silver wire counter electrode, and an Ag/AgCl (3 M KCl) reference electrode. The rotaxane **1a** (4.0 mg) was dissolved in dry dichloromethane (0.8 mL), sonicated for 5 min then diluted in acetonitrile (4 mL) to give a final concentration of 0.67 mM. Tetrabutylammonium hexafluorophosphate (0.10 M) was used as supporting electrolyte. Samples were degassed with argon for 5 min prior to measurement. In analogous conditions, ferrocene was used as internal reference.

### Synthetic procedures

The tetrabutylsuccinamide thread was prepared using a literature procedure [5]. 5-Ferrocenylisophthalic chloride was prepared using literature procedures [24–25]. *N*,*N’*-Dihexyl-1,4-butanediamide was prepared using a literature procedure [26].

#### Synthesis of rotaxane **1a**

A solution of *p*-xylylenediamine (**6**, 272 mg, 2 mmol) in chloroform (15 mL) and a solution of 5-ferrocenylisophthaloyl chloride (**5**, 774 mg, 2 mmol) in chloroform (15 mL) were simultaneously added with a syringe pump during 5 hours to a solution of tetrabutylsuccinamide thread **4a** (85 mg, 0.25 mmol) and dry triethylamine (0.84 mL, 6 mmol) in chloroform (50 mL). The solution was stirred at room temperature overnight. The reaction mixture was filtered through Celite and the filtrate was washed with hydrochloric acid solution (1 M, 10 mL), saturated sodium bicarbonate solution (10 mL), water (10 mL), and brine (10 mL). The organic layer was dried over MgSO_4_, filtered and the solvent removed in vacuo. Purification by silica gel column chromatography (DCM/ethyl acetate 9:1 (v/v)) afforded **1a** as beige solid (129 mg, 41% yield). ^1^H NMR (300 MHz, CDCl_3_) δ 8.66 (s, 2H, *H_e_*), 8.46 (d, *J* = 1.3 Hz, 4H, *H_d_*), 7.76 (t, *J* = 5.8 Hz, 4H, *H_f_*), 7.15 (s, 8H, *H_h_* + *H_i_*), 5.45 (br s, 4H, *H_g_*), 4.87 (t, *J* = 1.8 Hz, 4H, *H_a _*_or _*_b_*), 4.44 (t, *J* = 1.7 Hz, 4H, *H_b_*_ or _*_a_*), 4.09 (s, 10H, *H_c_*), 3.80 (br s, 4H, *H_g_*), 3.31–3.20 (m, 4H, *H_j’_*), 2.81–2.70 (m, 4H, *H_j_*), 1.58–1.50 (m, 4H, *H_k’_*), 1.40–1.30 (m, 4H, *H_l’_*), 1.28–1.20 (m, 4H, *H_k_*), 1.12 (s, 4H, *H_i_*), 0.97 (t, *J* = 7.3 Hz, 6H, *H_m’_*), 0.68–0.60 (m, 10H, *H_l_ + H_m_*); ^13^C NMR (151 MHz, CDCl_3_) δ 172.72, 165.46, 141.76, 138.71, 133.46, 129.25, 128.96, 119.70, 83.34, 69.75, 69.67, 66.89, 48.24, 46.63, 43.25, 30.79, 30.27, 28.15, 20.25, 19.83, 13.89, 13.77; HRMS–FD (*m*/*z*): [M]^+^ calcd for C_72_H_84_N_6_O_6_Fe_2_, 1240.5151; found, 1240.5152.

#### Synthesis of thread **4c**

Succinyl chloride (395 mg, 2.55 mmol) was added slowly to a cooled (0 °C) solution of 2,2-diphenylethanol (100.0 g, 5.1 mmol) in dry CH_2_Cl_2_ (50 mL). The brown solution was stirred at room temperature for 36 h under a N_2_ atmosphere. The solution was diluted with CH_2_Cl_2_ (50 mL) and an aqueous work up was performed with sat. NaHCO_3_ (2 × 100 mL), H_2_O (1 × 200 mL), and brine (1 × 100 mL). The organic extract was dried using Na_2_SO_4_, filtered, and the solvent was removed in vacuo. Finally, the product was purified by column chromatography using a solvent gradient (hexane/ethyl acetate 9:1 to 1:1, v/v) to obtain the pure product as white crystalline powder (622 mg, 51%). ^1^H NMR (300 MHz, CDCl_3_) δ 7.41–7.32 (m, 8H, *Ph*), 7.32–7.23 (m, 12H, *Ph*), 4.67 (d, *J* = 7.6 Hz, 4H, *CH*), 4.40 (t, *J* = 7.6 Hz, 2H, OC*H*_2_CH), 2.50 (s, 4H, *CH_2_*); ^13^C NMR (75 MHz, CDCl_3_) δ 172.1, 141.1, 128.6, 128.3, 126.9, 66.9, 49.8, 29.1; HRMS–ESI (*m*/*z*): [M + Na]^+^ calcd for C_32_H_30_O_4_Na, 501.20363; found, 501.20295.

### Crystal structure determination

Single crystals of rotaxane **1a** (C_36_H_42_FeN_3_O_3_) were obtained by slow evaporation of a dichloromethane solution. A suitable crystal was mounted on a cryoloop with paratone^®^-N oil on an AFC11 partial Chi goniometer. The data were collected at 120 K on Rigaku FRX^®^ high flux rotating anode with a Pilatus 200K hybrid pixel detector. Using Olex2 [27], the structure was solved with the ShelXT [28] structure solution program using Intrinsic Phasing and refined with the ShelXL [29] refinement package using the full least-squares correlation matrix. The non-hydrogen atoms were located in successive difference Fourier maps and refined with anisotropic thermal parameters on F^2^. All hydrogen atoms were generated theoretically at the specific atoms positions and refined isotropically with fixed thermal factors.

Crystal data for rotaxane **1a** C_36_H_42_FeN_3_O_3_ (*M* = 620.57 g/mol): triclinic, space group *P*-1 (no. 2), *a* = 9.5366(5) Å, *b* = 11.6279(7) Å, *c* = 14.9396(9) Å, α = 99.002(5), β = 108.001(5), *γ* = 90.007(4), *V* = 1554.09(16) Å^3^, *Z* = 2, *T* = 120 K, μ(Cu Kα) = 4.208 mm^−1^, *D*_calc_ = 1.326 g/cm^3^, 14633 reflections measured (9.124° ≤ 2Θ ≤ 111.358°), 3928 unique (*R*_int_ = 0.0450, R_sigma_ = 0.0402) which were used in all calculations. The final *R*_1_ was 0.0454 (I > 2σ(I)) and *wR*_2_ was 0.1180 (all data). CCDC Deposition Number 1968472.

### Molecular modelling

Monte Carlo conformational searches with the MMFF force field were carried out with the Spartan ‘18 software [30]. For each structure 2025 conformers were examined (see text) and the obtained conformers were ordered by relative energy to obtain the most stable one.

## Supporting Information

File 1 Further experimental details and NMR spectra of new compounds.

File 2 X-ray data of rotaxane **1a**.
