# Supplementary material for: Five-component, one-pot synthesis of an electroactive rotaxane comprising a bisferrocene macrocycle
Source: Beilstein J Org Chem. 2020 Jun 30;16:1564–71. doi: 10.3762/bjoc.16.128 (PMC7356209; doi:10.3762/bjoc.16.128)
Supplement: File 1 — Further experimental details and NMR spectra of new compounds. [file Beilstein_J_Org_Chem-16-1564-s001.pdf]

BEILSTEIN JOURNAL OF ORGANIC CHEMISTRY

Supporting Information

for

Five-component, one-pot synthesis of an electroactive rotaxane comprising a bisferrocene macrocycle

Natalie Lagesse, Luca Pisciottani, Maxime Douarre, Pascale Godard, Brice Kauffmann, Vicente Martí-Centelles and Nathan D. McClenaghan

Beilstein J. Org. Chem. **2020,** *16,* 1564–1571. doi:10.3762/bjoc.16.128

Further experimental details and NMR spectra of new compounds

License and Terms: This is a supporting information file under the terms of the Creative Commons Attribution License (http://creativecommons.org/licenses/by/4.0). Please note that the reuse, redistribution and reproduction in particular requires that the authors and source are credited.
The license is subject to the *Beilstein Journal of Organic Chemistry* terms and conditions: (https://www.beilstein-journals.org/bjoc)

Electrochemistry

Cyclic voltammetry was carried out in a Metrohm Autolab PGSTAT302N potentiostat using a three-electrode cell, with a glassy carbon working electrode, silver wire counter electrode and Ag/AgCl (3M KCl) reference electrode. The glassy carbon working electrode was polished in a figure of eight fashion using 1, 0.3 and 0.05 gamma alumina on a microcloth pad (Buehler, UK) (for 3.3 and 5 min, respectively). The electrodes were then sonicated in 1:1 ethanol/water for 5 min before being finally washed in stream of dry tetrahydrofuran. The counter electrode was a silver wire which was cleaned by annealing with a Bunsen burner. A Ag/AgCl reference electrode, stored in 3 M KCl, and washed thoroughly before use was used initially, before changing to a Ag wire pseudo-reference electrode, heat-annealed with a Bunsen burner and quenched with dry DCM.

The supporting electrolyte was a solution of 0.1M $TBAPF_6$ containing 1 mM ferrocene (to act as an internal reference), dissolved in dry CH_3CN, and degassed with argon for 5 min. The rotaxane **1a** (4.0 mg) was dissolved in dry dichloromethane (0.8 mL), sonicated for 5 min, then diluted in acetonitrile (4 mL) to give a final concentration of 0.67 mM. Tetrabutylammonium hexafluorophosphate (0.10 M) was used as supporting electrolyte. Samples where degassed with argon for 5 min prior to measurement. In analogous conditions ferrocene was used as internal reference.

NMR spectra

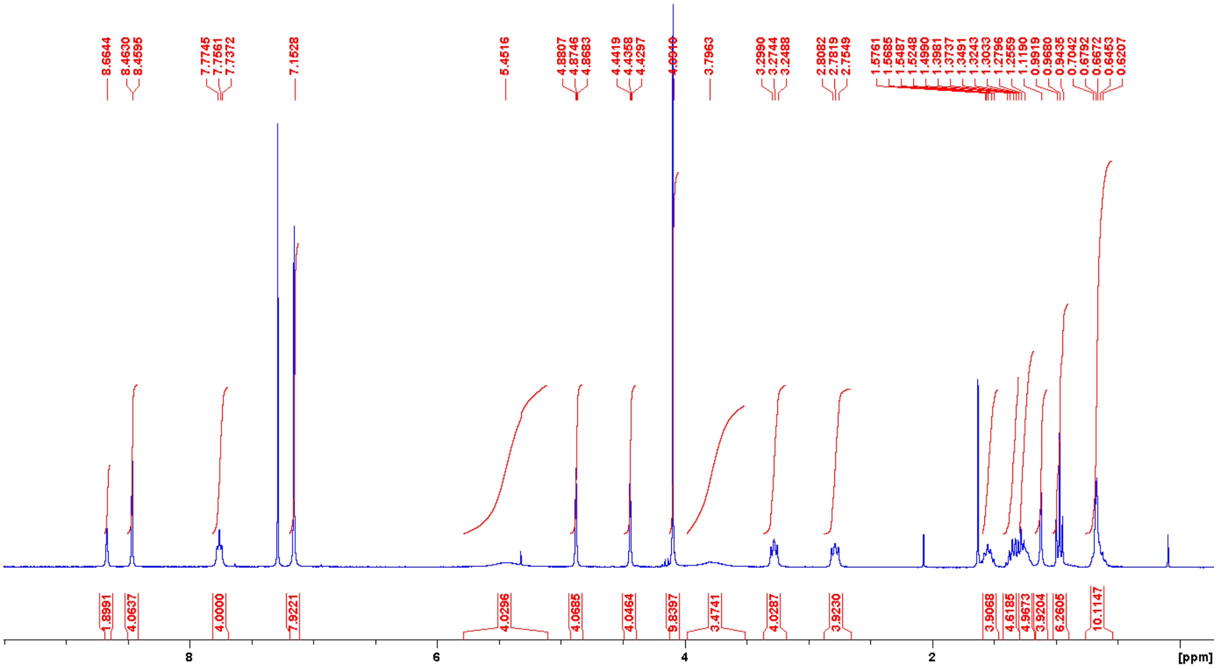

Figure S1: ^{1}H-NMR of ferrocene-containing clipped [2]rotaxane **1a** (300 MHz in $CDCl_3$).

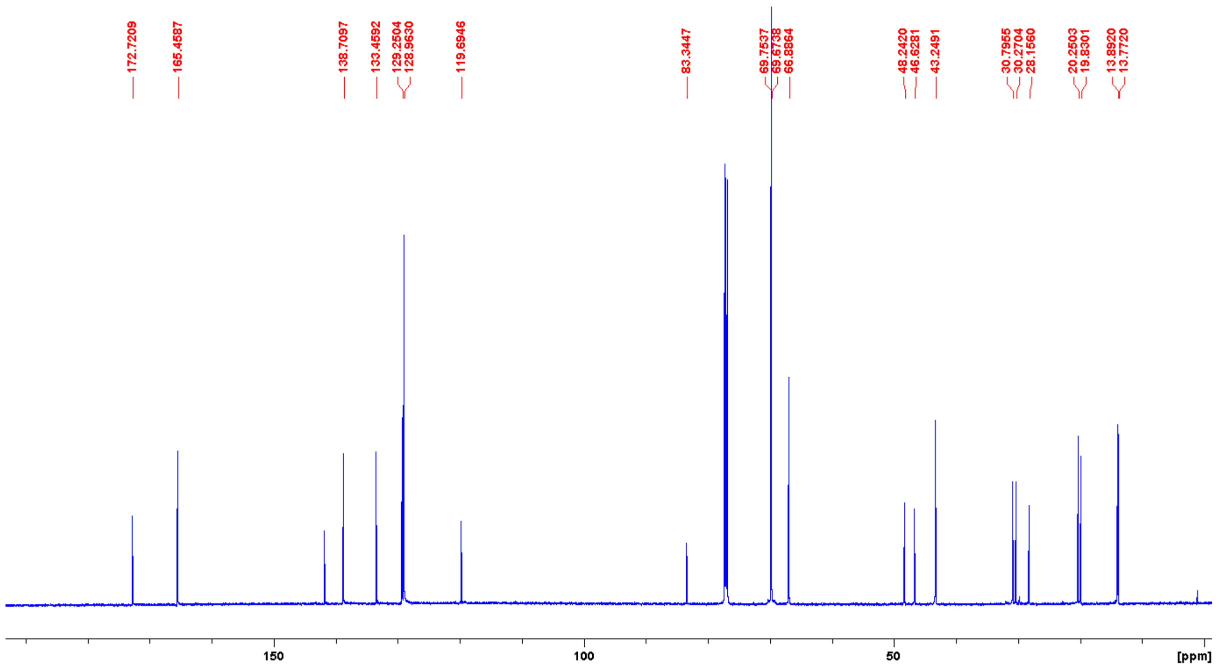

Figure S2: ^{13}C-NMR of ferrocene-containing clipped [2]rotaxane **1a** (150 MHz in $CDCl_3$).

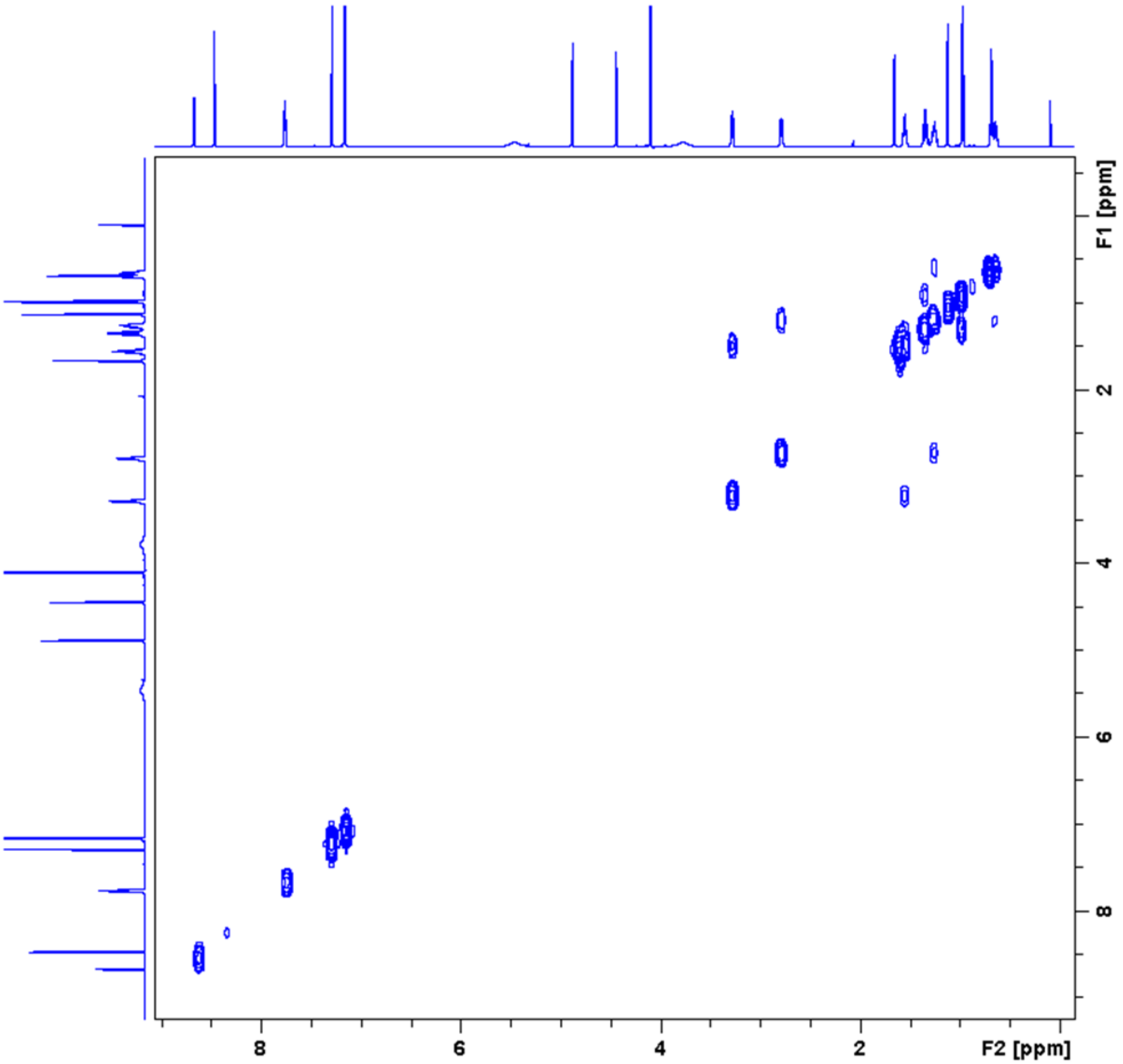

Figure S3: ^{1}H-^{1}H COSY NMR of ferrocene-containing clipped [2]rotaxane **1a** (600 MHz in $CDCl_3$).

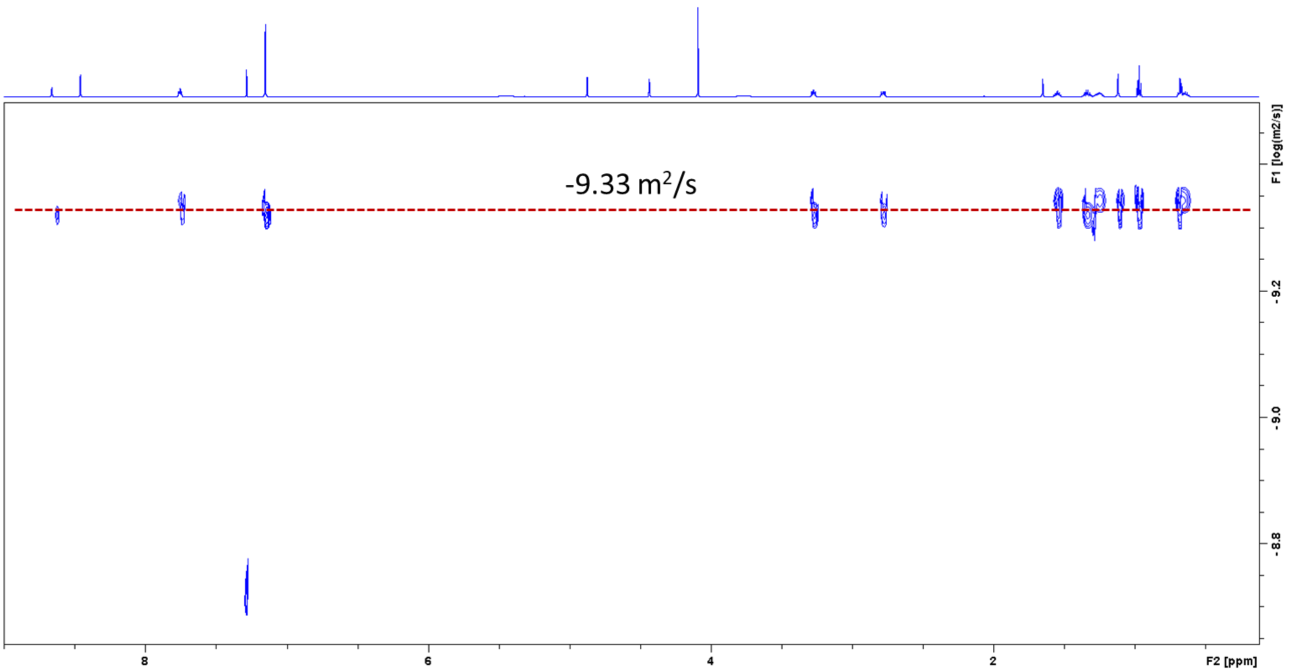

Figure S4: ^{1}H DOSY NMR of ferrocene-containing clipped [2]rotaxane **1a** (600 MHz in $CDCl_3$).

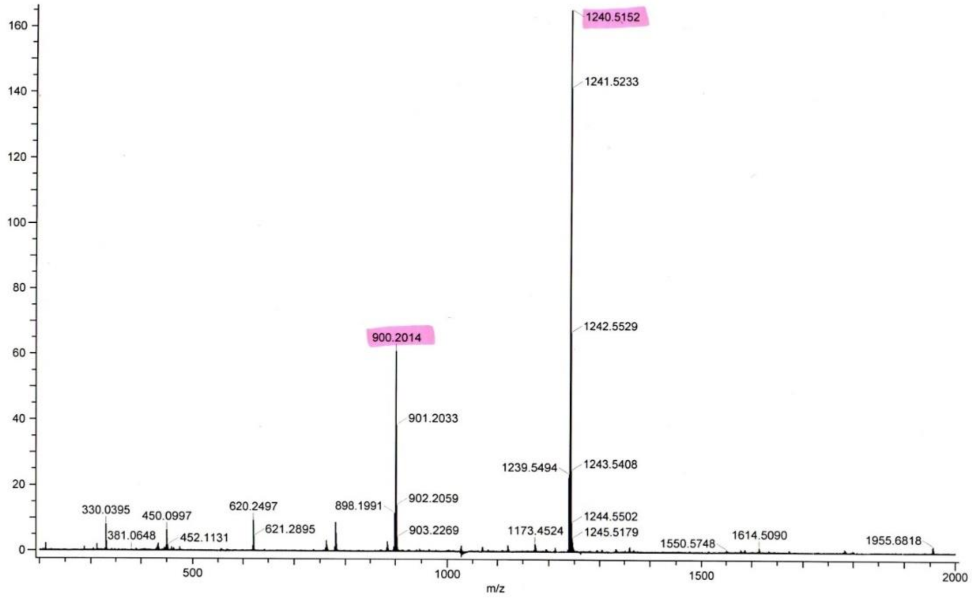

Figure S5: Mass spectrum (FD) of ferrocene-containing clipped [2]rotaxane **1a**.

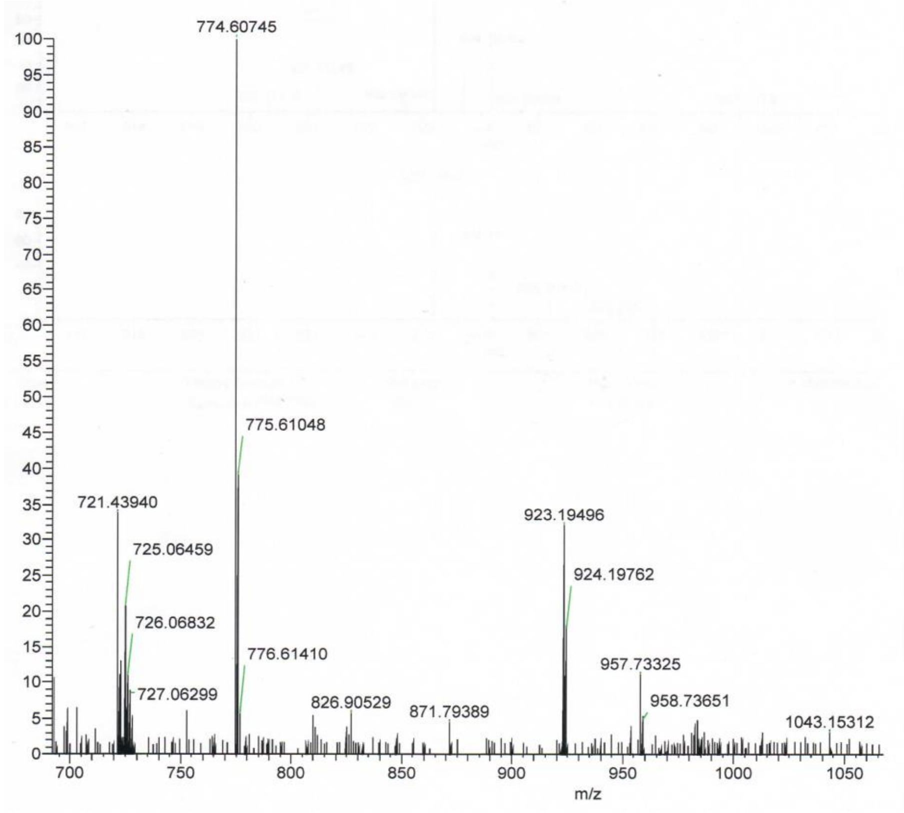

Figure S6: Mass spectrum (FD) of ferrocene macrocyle **2**.

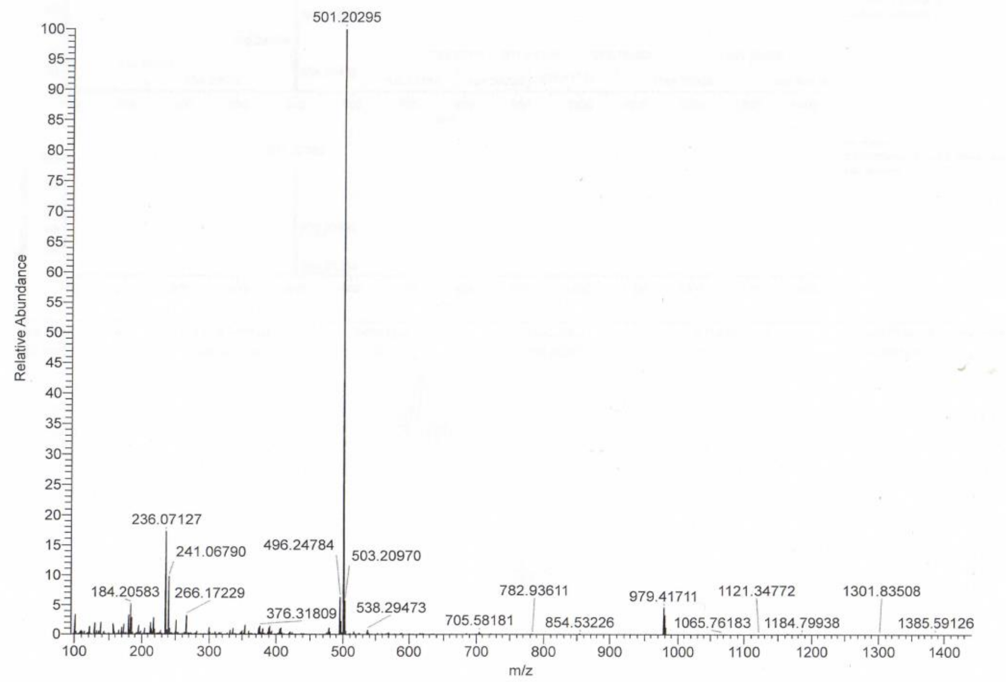

Figure S7: Mass spectrum (ESI) of double ester thread **4c**.

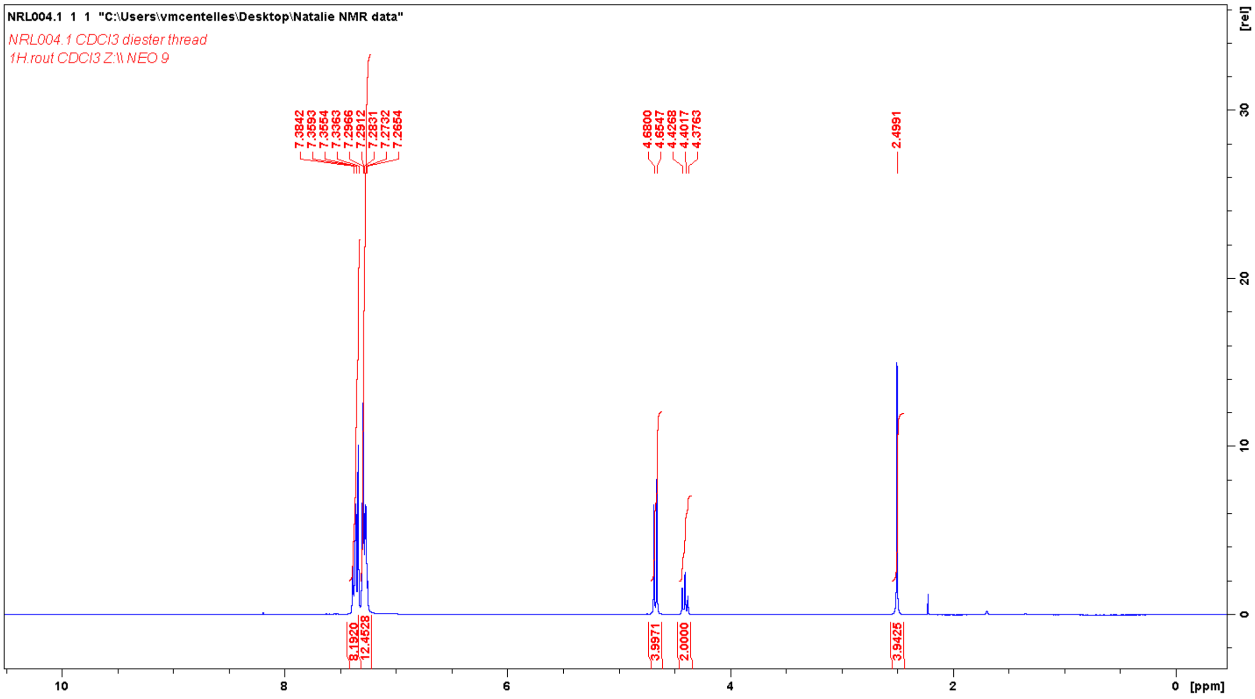

Figure S8: ^{1}H NMR of double ester thread **4c** (300 MHz in $CDCl_3$).

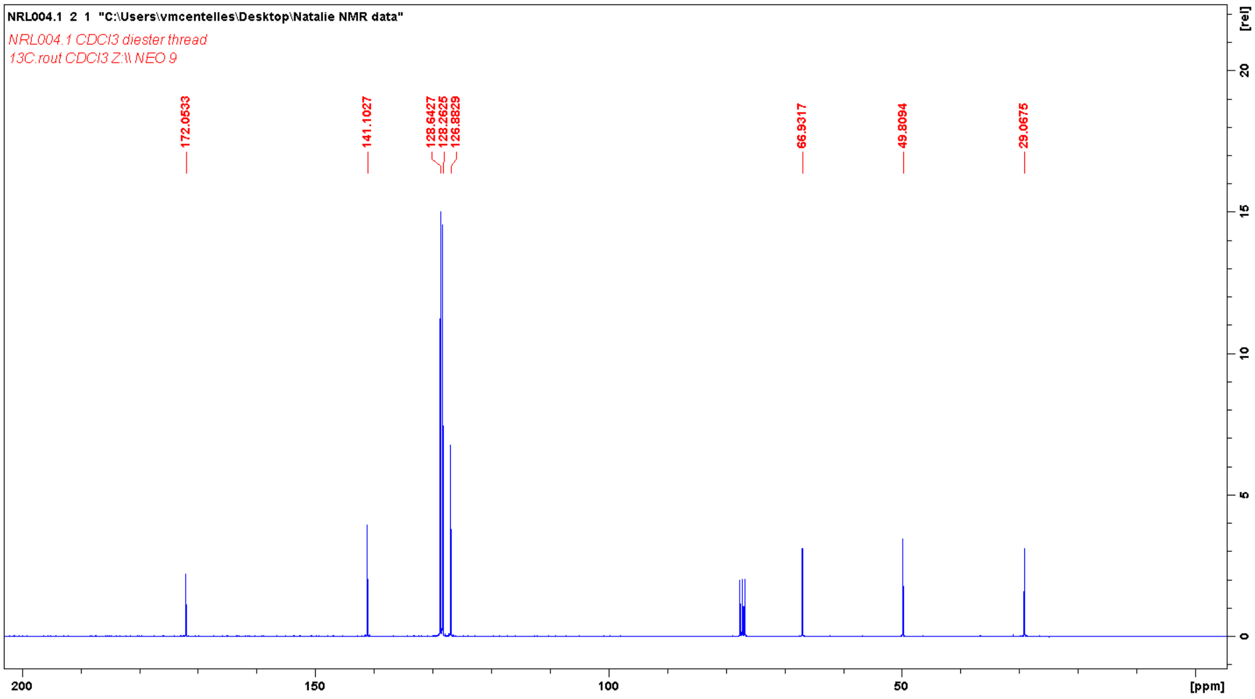

Figure S9: ^{13}C NMR of double ester thread **4c** (75 MHz in $CDCl_3$).

Synthesis attempt of rotaxane 1b

A solution of *p*-xylylenediamine (**6**, 68 mg, 0.5 mmol) in chloroform (20 mL) and a solution of 5-ferrocenylisophthaloyl chloride (**5**, 200 mg, 0.5 mmol) in chloroform (20 mL) were simultaneously added with a syringe pump over 5 hours to a solution of unsymmetrical ester amide thread **4b** (122 mg, 0.25 mmol) and dry triethylamine (0.35 mL, 2.6 mmol) in chloroform (20 mL), and the solution was stirred at room temperature overnight. After this time, DCM (25 mL) was added to the reaction mixture followed by filtration through Celite. The filtrate was washed with sodium carbonate solution 10% (2 × 50 mL). The organic layer was dried over Na_2SO_4, filtered, and the solvent was removed in vacuo. Analysis of the crude ^{1}H NMR allowed estimation of a < 5% yield of the rotaxane.

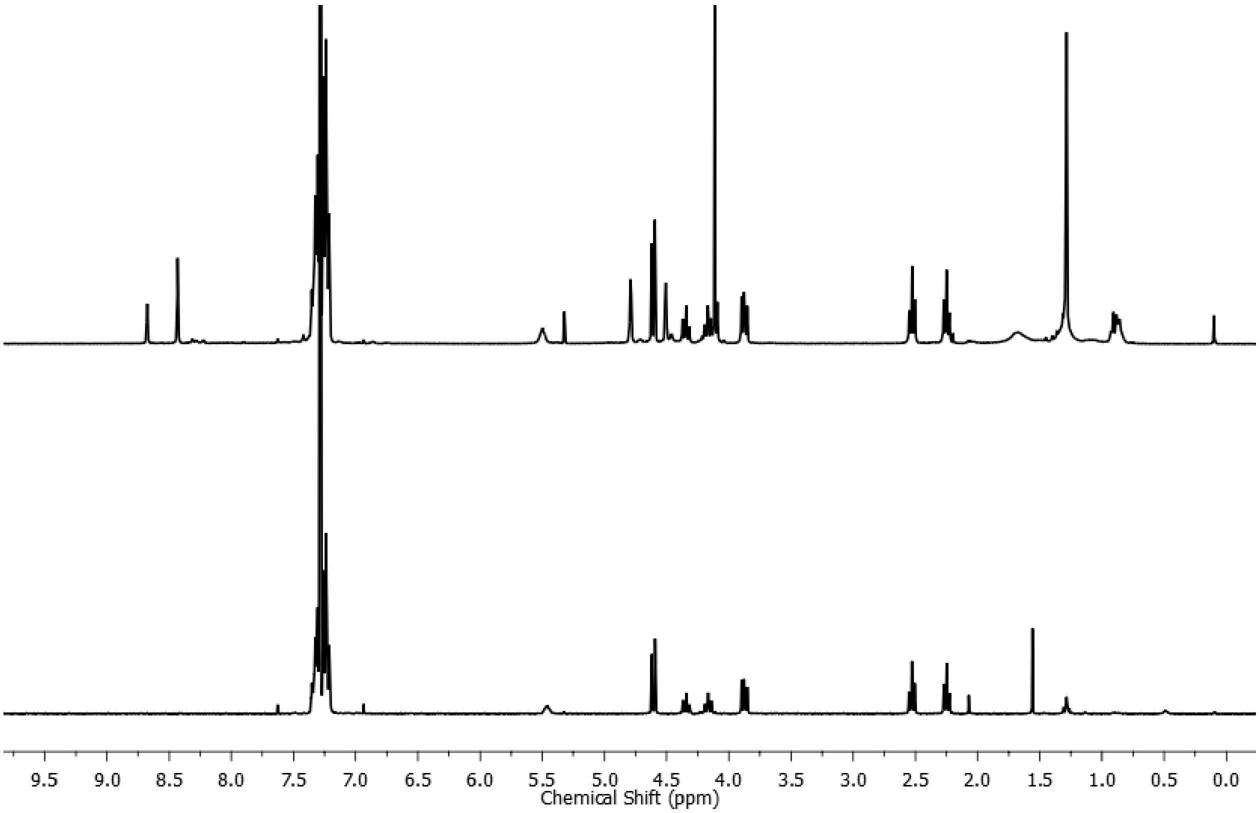

Figure S10: ^{1}H NMR of ester amide thread **4b** (bottom) and crude reaction mixture of the rotaxane formation reaction (top) (300 MHz in $CDCl_3$).

Synthesis attempt of rotaxane 1c

A solution of *p*-xylylenediamine (**6**, 17 mg, 0.12 mmol) in chloroform (10 mL) and a solution of 5-ferrocenylisophthaloyl chloride (**5**, 50 mg, 0.12 mmol) in chloroform (10 mL) were simultaneously added with a syringe pump during 4 hours to a solution of thread **4c** (30 mg, 0.06 mmol) and dry triethylamine (86 µL, 0.6 mmol) in chloroform (10 mL). The reaction mixture was stirred at room temperature overnight. After this time, DCM (25 mL) was added to the suspension followed by filtration through Celite. The filtrate was washed with sodium carbonate solution 10% (2 × 25 mL). The organic layer was dried over Na_2SO_4, filtered, and the solvent removed in vacuo. It was not possible to identify any proton signals corresponding to the rotaxane in the crude ^{1}H NMR.

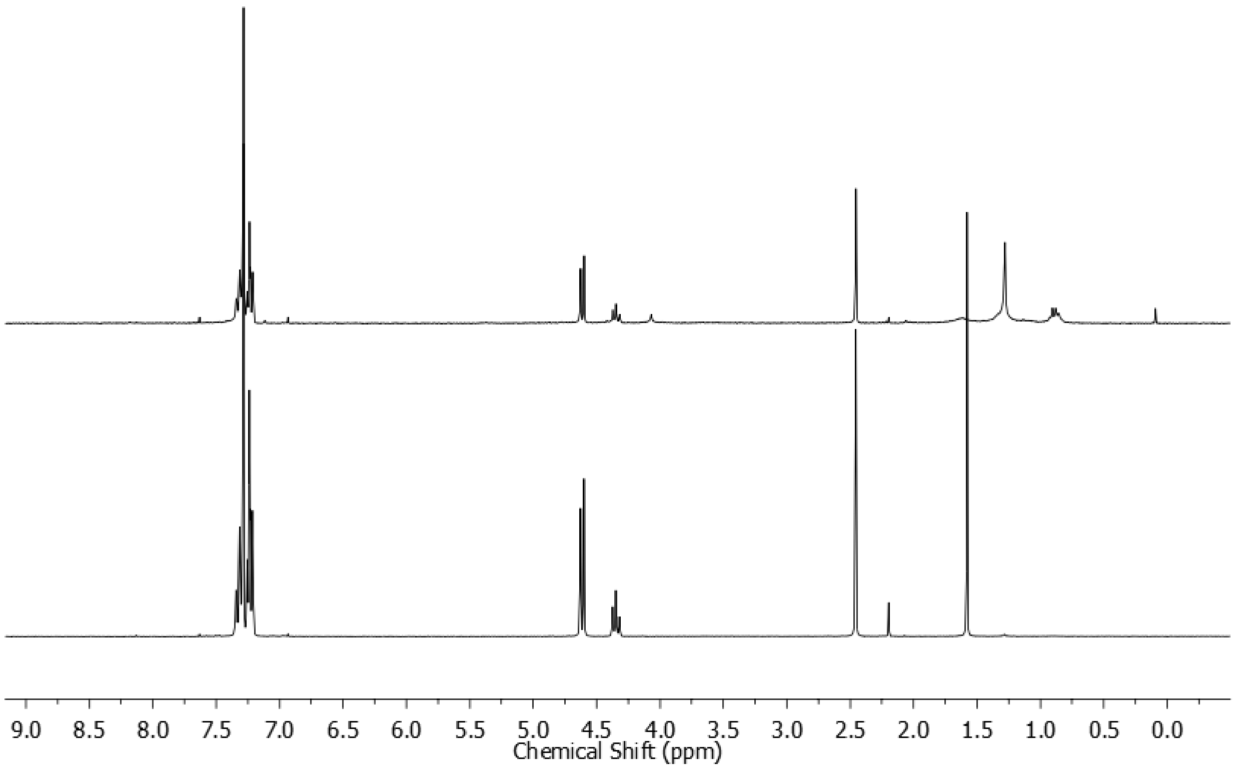

Figure S11: ^{1}H NMR of double ester thread **4c** (bottom) and crude reaction mixture of the rotaxane formation reaction (top) (300 MHz in $CDCl_3$).

Synthesis attempts of macrocycle 2

Templated macrocyclization

A solution of *p*-xylylenediamine (**6**, 17.6 mg, 0.129 µmol) in chloroform (10 mL) and a solution of 5-ferrocenylisophthaloyl chloride (**5**, 50.0 mg, 0.129 µmol) in chloroform (15 mL) were simultaneously added with a syringe pump during 3 hours to a solution of the *N*,*N'*-dihexyl-1,4-butanediamide thread **3** (36.7 mg, 0.129 µmol) and dry triethylamine (49 µL, 0.323 µmol) in chloroform (10 mL). The reaction mixture was stirred at room temperature overnight. The reaction mixture was diluted with CH_2Cl_2 (25 mL) and filtered through Celite. The filtrate was washed with saturated $NaHCO_3$ (10 mL) and the solvent removed in vacuo. Purification by silica gel column chromatography (CH_2Cl_2/MeOH 0% to 10%, v/v) afforded impure macrocycle **2** as yellow solid (17.0 mg, < 29% yield).

Macrocycle dethreading

Rotaxane **1a** (30 mg, 0.024 mmol) was suspended in DMSO-d_6 (2.5 mL) and heated in a sand bath (160 °C, 25 hours) with stirring. As the temperature of the suspension exceeded 150 °C, the solid began to dissolve. After 4 hours it was fully dissolved and remained dissolved upon cooling to rt. After 6 hours, the color of the solution began to darken and after 25 hours the solution was dark brown and black material precipitated upon cooling. This reaction was monitored by ^{1}H NMR until completion (Figure S12). Concentration of species was estimated from the integrals of the ^{1}H NMR spectra (Figure S13). The analysis of the ^{1}H NMR at 22.5 h of reaction showed the presence of free macrocycle (Figure S14). In this reaction mixture the macrocycle could be identified by HRMS–FD (*m/z*): $[M + Na]^+$ calcd for $C_{52}H_{44}N_4O_4Fe_2Na$, 923.19536; found, 923.19496.

A similar experiment was performed, where rotaxane **1a** (25.7 mg, 0.023 mmol) was suspended in DMSO-d_6 (0.75 mL) and heated for a shorter time (6 h). Ice was added to the resulting orange solution and the orange precipitate was filtered via gravity filtration, washed with water (10 mL), and left to dry. The solid

was then dissolved in dichloromethane, dried over Na_2SO_4, filtered and the solvent was removed in vacuo. The resulting solid was analyzed by ^{1}H NMR showing only starting material.

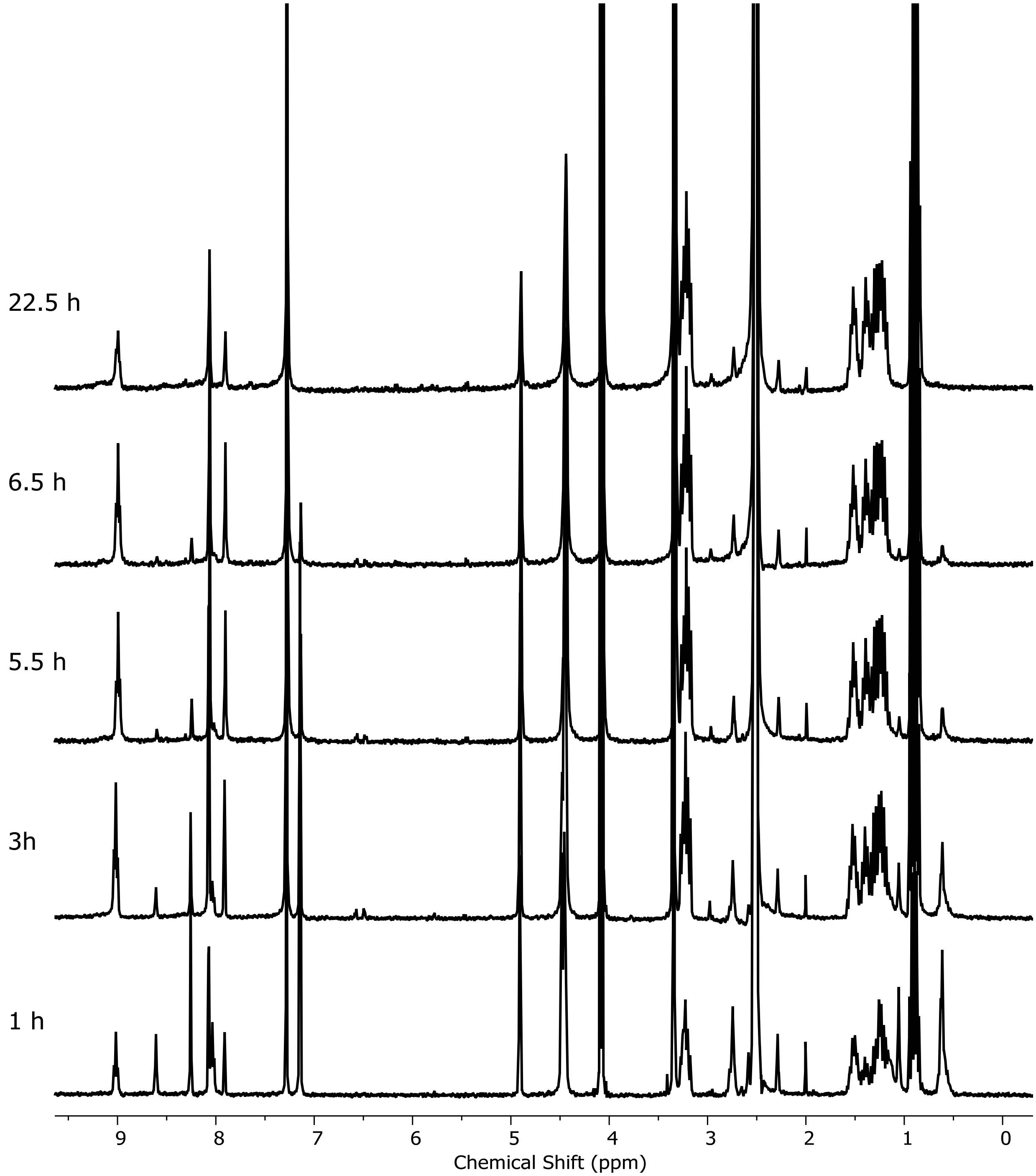

Figure S12: ^{1}H NMR of the dethreading reaction of rotaxane **1a** (300 MHz in DMSO-d_6) at 1 h, 3 h, 5.5 h, 6.5 h, and 22.5 h.

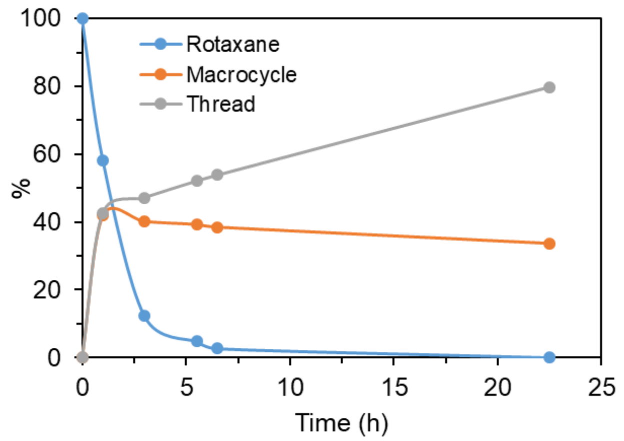

Figure S13: Relative distribution of species in the dethreading reaction of rotaxane **1a** estimated from the crude ^{1}H NMR integrals.

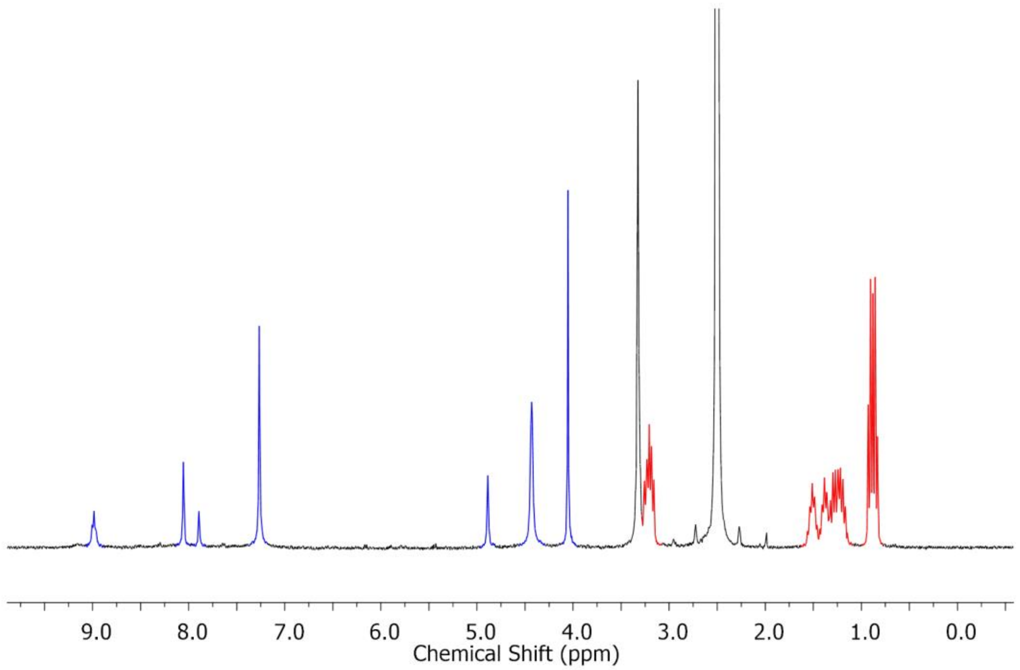

Figure S14: ^{1}H NMR of the dethreading reaction of rotaxane **1a** (300 MHz in DMSO-d_6) after 22.5 h of heating 160 °C showing NMR signals of free macrocycle **2** (blue) and free thread **4a** (red).

X-Ray data

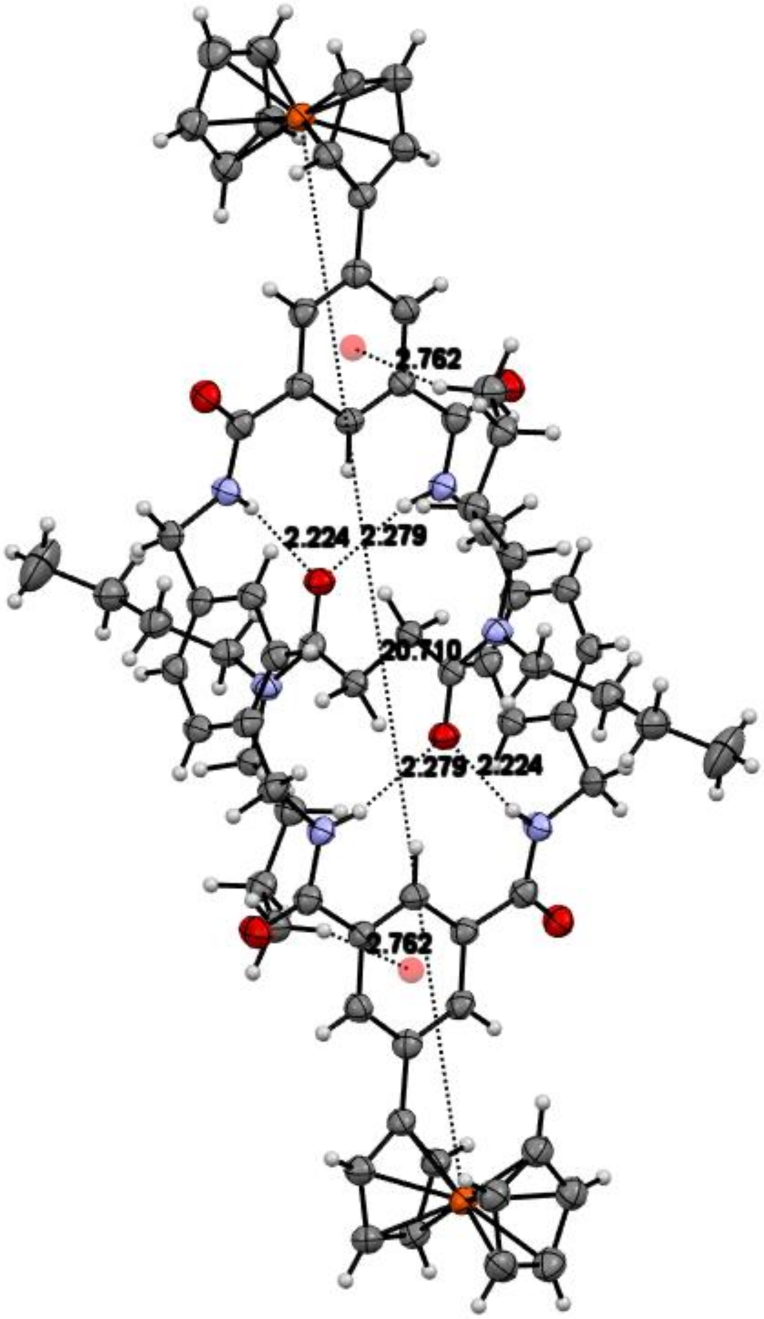

Figure S15: Selected distances in the solid-state structure of rotaxane **1a**: macrocycle-thread hydrogen bond distances, Fe–Fe distance, and thread CH_3 to isophthalamide centroid.

Table S1: Crystal data and structure refinement for rotaxane **1a**.

CCDC Deposition Number	1968472
Empirical formula	$C_{36}H_{42}FeN_3O_3$
Formula weight	620.57
Temperature/K	120
Crystal system	triclinic
Space group	P-1
a/Å	9.5366(5)
b/Å	11.6279(7)
c/Å	14.9396(9)
α/°	99.002(5)
β/°	108.001(5)
γ/°	90.007(4)
Volume/Å^3	1554.09(16)
Z	2
ρ_{calc}g/cm^3	1.326
μ/mm^{-1}	4.208
F(000)	658.0
Crystal size/mm^3	0.05 × 0.01 × 0.01
Radiation	CuKα (λ = 1.54184)
2Θ range for data collection/°	9.124 to 111.358
Index ranges	$-10 \leq h \leq 9$, $-12 \leq k \leq 12$, $-15 \leq l \leq 15$
Reflections collected	14633
Independent reflections	3928 [R_{int} = 0.0450, R_{sigma} = 0.0402]
Data/restraints/parameters	3928/0/391
Goodness-of-fit on F^2	1.041
Final R indexes [I>=2σ (I)]	R_1 = 0.0454, wR_2 = 0.1116
Final R indexes [all data]	R_1 = 0.0556, wR_2 = 0.1180
Largest diff. peak/hole / e Å^{-3}	0.28/-0.41

XYZ coordinates of the optimized structures

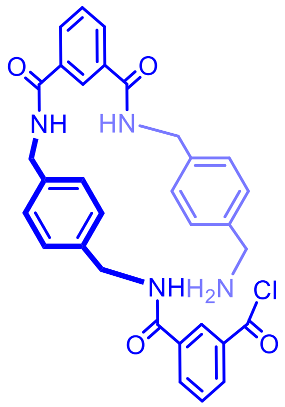

H	-1.907450	0.385310	4.976331
C	-0.944136	0.002150	4.646760
C	1.538938	-0.967625	3.829695
C	-0.709972	-1.375520	4.620357
C	0.046272	0.888962	4.221839
C	1.284509	0.404769	3.785625
C	0.532451	-1.870140	4.201975
H	-1.516512	-2.039524	4.919922
H	-0.154469	1.959003	4.211855
H	2.520458	-1.341950	3.556194
C	0.815804	-3.337860	4.087942
C	2.277286	1.368148	3.234104
O	1.874028	-3.666332	3.547737
O	2.229411	2.546182	3.571490
N	3.164802	0.871603	2.299392
H	3.032565	-0.068026	1.927545
H	-5.204034	0.297788	-5.820528
C	-4.514129	0.386685	-4.984291
C	-2.730578	0.615151	-2.843417
C	-4.748503	-0.326602	-3.807515
C	-3.388827	1.209304	-5.092955
C	-2.481008	1.312420	-4.030998
C	-3.869391	-0.195850	-2.729619
H	-5.615018	-0.980426	-3.729671
H	-3.214154	1.761465	-6.015258
H	-2.038334	0.687337	-2.011663
C	-4.107603	-0.983909	-1.492389
C	-1.283354	2.188420	-4.188174
O	-4.534146	-2.129145	-1.563383
O	-1.284656	3.091091	-5.016602
N	-3.778744	-0.348219	-0.312844
H	-3.467708	0.611130	-0.347184
N	-0.209582	1.912548	-3.370021
H	-0.149914	1.013938	-2.911643
C	3.966771	1.760552	1.485901
H	4.175664	2.694150	2.020055
H	4.926260	1.263364	1.306589
C	1.018650	2.670075	-3.466339
H	0.792385	3.731065	-3.624088

H	1.563403	2.320925	-4.350962
C	-3.364616	-1.119850	0.839191
H	-3.525656	-0.508894	1.733658
H	-3.980315	-2.021626	0.925067
C	3.270098	2.048285	0.177412
C	1.839205	2.481872	-2.217030
C	2.269467	3.027343	0.111366
C	3.552465	1.292655	-0.968480
C	2.842669	1.506157	-2.154251
C	1.560110	3.240521	-1.072374
H	2.024523	3.614906	0.995578
H	4.313628	0.516001	-0.937420
H	3.070467	0.895997	-3.025737
H	0.776036	3.995070	-1.093544
C	-1.898777	-1.464069	0.709909
C	0.815808	-2.080965	0.233755
C	-1.488958	-2.792065	0.539447
C	-0.932321	-0.449762	0.671907
C	0.404543	-0.755621	0.418156
C	-0.143086	-3.098350	0.315592
H	-2.222096	-3.597169	0.545350
H	-1.214258	0.591293	0.811008
H	1.128579	0.051980	0.359678
H	0.144771	-4.138686	0.177909
C	2.258333	-2.398996	-0.061917
H	2.596656	-1.786851	-0.905310
H	2.363536	-3.445215	-0.371698
N	3.112867	-2.152749	1.098733
H	4.055082	-2.470850	0.868429
H	2.806090	-2.803781	1.835972
Cl	-0.342284	-4.498983	4.697882

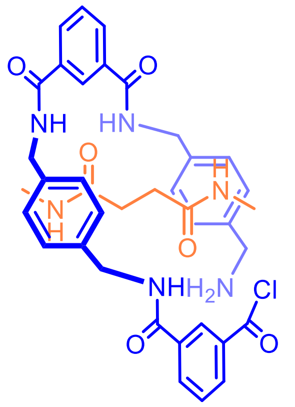

H	5.361691	-5.628340	1.844246
C	4.750336	-4.728498	1.861959
C	3.193530	-2.411463	1.925563
C	5.056998	-3.656800	1.017271
C	3.664113	-4.645958	2.732974
C	2.872920	-3.494427	2.754142
C	4.282700	-2.487589	1.045379
H	5.910538	-3.758419	0.351957

H	3.433089	-5.478442	3.395303
H	2.600750	-1.502099	1.973514
C	4.581452	-1.310449	0.163533
C	1.728878	-3.420408	3.700856
O	3.940251	-0.274207	0.355401
O	1.774523	-4.014855	4.770529
N	0.662339	-2.661409	3.282982
H	0.538494	-2.530087	2.278923
H	-3.453195	7.574545	-1.963296
C	-3.224206	6.526917	-1.784830
C	-2.660305	3.813919	-1.336544
C	-2.583986	5.772846	-2.766220
C	-3.576605	5.928591	-0.577025
C	-3.288755	4.575761	-0.343894
C	-2.292060	4.418386	-2.545723
H	-2.316387	6.241885	-3.711862
H	-4.082977	6.518379	0.185544
H	-2.493260	2.752745	-1.183872
C	-1.629402	3.665511	-3.645764
C	-3.707647	3.993951	0.958814
O	-1.738026	4.059858	-4.802795
O	-4.652489	4.486120	1.567535
N	-0.881666	2.560083	-3.294793
H	-0.584861	2.471238	-2.322947
N	-2.965493	2.935957	1.447821
H	-2.047861	2.766396	1.048972
C	-0.517506	-2.503699	4.099998
H	-1.162638	-3.368886	3.910795
H	-0.250051	-2.516751	5.162770
C	-3.197384	2.514989	2.811955
H	-4.276400	2.419922	2.985846
H	-2.845116	3.313599	3.476528
C	-0.051925	1.935156	-4.300117
H	-0.598072	1.873393	-5.249348
H	0.808143	2.592847	-4.474252
C	-1.220183	-1.212180	3.765744
C	-2.505447	1.220393	3.137834
C	-2.518389	-1.209919	3.242535
C	-0.572634	0.015008	3.973049
C	-1.204138	1.218767	3.653757
C	-3.154109	-0.004808	2.929763
H	-3.044002	-2.146682	3.070840
H	0.436077	0.033541	4.382525
H	-0.677585	2.155931	3.822502
H	-4.164314	-0.024249	2.525160
C	0.399166	0.560861	-3.887388
C	1.232965	-2.024329	-3.115973
C	1.707944	0.339901	-3.444643
C	-0.487775	-0.522800	-3.946563
C	-0.075388	-1.802592	-3.564386
C	2.121568	-0.940996	-3.072846
H	2.418205	1.161975	-3.389239
H	-1.507947	-0.368896	-4.293355
H	-0.784684	-2.624958	-3.620784
H	3.144468	-1.093881	-2.737038
C	1.685462	-3.403586	-2.709309
H	2.487476	-3.728314	-3.381626
H	0.864855	-4.122068	-2.817396
N	2.159653	-3.433990	-1.329060
H	1.384517	-3.147855	-0.716433
H	2.344917	-4.402242	-1.070887
Cl	5.821583	-1.434832	-1.067781

C	0.605914	0.091737	0.242741
H	0.778102	-0.020925	1.317643
H	1.347155	-0.520902	-0.278698
C	-0.796318	-0.367956	-0.118467
H	-1.023197	-0.127409	-1.160999
H	-1.537066	0.148526	0.497520
C	-0.946637	-1.859022	0.074085
C	0.835506	1.536532	-0.124337
O	-0.058786	-2.577819	0.528675
O	-0.058915	2.283012	-0.515070
N	-2.184035	-2.331068	-0.300248
H	-2.808059	-1.707833	-0.798812
N	2.141301	1.932211	0.046602
H	2.846583	1.201897	0.177865
C	2.592283	3.240817	-0.347170
H	3.540602	3.447747	0.153673
H	2.735540	3.248679	-1.430913
H	1.850787	3.996258	-0.073794
C	-2.499140	-3.735579	-0.261367
H	-2.068236	-4.213537	-1.145058
H	-3.585435	-3.849215	-0.270577
H	-2.082682	-4.193556	0.639876
